# Virtual monoenergetic imaging predicting Ki-67 expression in lung cancer

**DOI:** 10.1038/s41598-023-30974-5

**Published:** 2023-03-07

**Authors:** Peipei Dou, Hengliang Zhao, Dan Zhong, Yingliang Hu, Bin Liu, Haiyan Zhang, Aihong Cao

**Affiliations:** 1 Department of Radiology, The Second Affiliated Hospital of Xuzhou Medical University, Xuzhou, Jiangsu Province People’s Republic of China; 2 Xuzhou Medical University, Xuzhou, Jiangsu Province People’s Republic of China; 3 The Affiliated Hospital of Xuzhou Medical University, Xuzhou, Jiangsu Province People’s Republic of China

**Keywords:** Cancer, Health care, Oncology

## Abstract

This study aimed to optimize slope and energy levels for evaluating Ki-67 expression in lung cancer using virtual monoenergetic imaging and compare the predictive efficiency of different energy spectrum slopes (λHU) for Ki-67. Forty-three patients with primary lung cancer confirmed via pathological examination were enrolled in this study. They underwent baseline arterial-phase (AP) and venous-phase (VP) energy spectrum computed tomography (CT) scanning before surgery. The CT values were 40–190 keV, with 40–140 keV indicating pulmonary lesions at AP and VP, and *P* < 0.05 indicating a statistically significant difference. An immunohistochemical examination was conducted, and receiver operating characteristic curves were used to analyze the prediction performance of λHU for Ki-67 expression. SPSS Statistics 22.0 (IBM Corp., NY, USA) was used for statistical analysis, and *χ*^2^, *t*, and Mann–Whitney U tests were used for quantitative and qualitative analyses of data. Significant differences were observed at the corresponding CT values of 40 keV (as 40-keV is considered the best for single-energy image for evaluating Ki-67 expression) and 50 keV in AP and at 40, 60, and 70 keV in VP between high- and low-Ki-67 expression groups (*P* < 0.05). In addition, the λHU values of three-segment energy spectrum curve in both AP and VP were quite different between two groups (*P* < 0.05). However, the VP data had greater predictive values for Ki-67. The areas under the curve were 0.859, 0.856, and 0.859, respectively. The 40-keV single-energy sequence was the best single-energy sequence to evaluate the expression of Ki-67 in lung cancer and to obtain λHU values using the energy spectrum curve in the VP. The CT values had better diagnostic efficiency.

## Introduction

As per the estimates of the global cancer incidence and mortality published by the International Agency for Research on Cancer in 2020, lung cancer (11.4%) is the second most common cancer after breast cancer (11.7%), resulting in 18.0% of all cancer deaths^1^. As most patients do not know about the nature of the tumor in the early stage of lung cancer, its prognosis becomes difficult and delayed. Studies on molecular biology focusing on the analysis and characterization of proteins and genes involved in cancer development can improve our knowledge of prognostic factors^2^. Among the new biological markers that can become useful prognostic factors for lung cancer, Ki-67 is a nuclear protein strongly associated with tumor cell proliferation and growth, which is widely used in routine pathological examinations as a proliferation marker^3–5^. Some studies have suggested an association between Ki-67 and poor survival in patients with lung cancer^6^. At present, immunohistochemistry (IHC) for Ki-67 expression is the gold standard^7^.However, due to the limitations of their own physical conditions, some patients cannot undergo biopsy, and materials from the preoperative pathology bronchial endoscopy may not allow immunohistochemical detection to reflect the panorama of the tumor accurately^8,9^. All the aforementioned factors limit preoperative diagnosis and the application of IHC.

In recent years, dual-energy computed tomography (DECT) has offered several postprocessing techniques to reflect the distinctive spectral curve of x-ray of different lesions and tissues, showing its great potential for use in the diagnosis, level of identification, and prediction of immune differentiation indicators of various tumors^10–13^. Besides providing material-specific information (e.g., iodine quantification), dual-energy CT datasets may also be used to synthesize virtual monochromatic images across a wide x-ray energy range (40–190 keV)^14^. Virtual monoenergetic imaging (VMI) reconstructed using DECT datasets allows the reconstruction of an image series at a desired hypothetical energy level (measured in keV)^15^. Furthermore, in interrogating the attenuation characteristics of different materials at different x-ray energies, virtual monochromatic images can generate tissue-specific spectral attenuation curves based on the unique K-edge characteristics of materials with different elemental compositions^16^. This technique has so far mainly been used for reducing metal artifacts, improving visualization of soft-tissue lesions^17^, and optimally visualizing intravascular iodine during CT angiography^18^. However, no studies to date have been specifically designed to assess whether Ki-67 expression in lung cancer may be predicted at a certain energy or slope level using an energy spectrum curve via VMI reconstructions.

Thus, this study aimed to investigate VMI in predicting Ki-67 expression in lung cancer and define the optimum slope level based on the energy spectrum curve.

## Methods

### Clinical data

**Figure 1:**
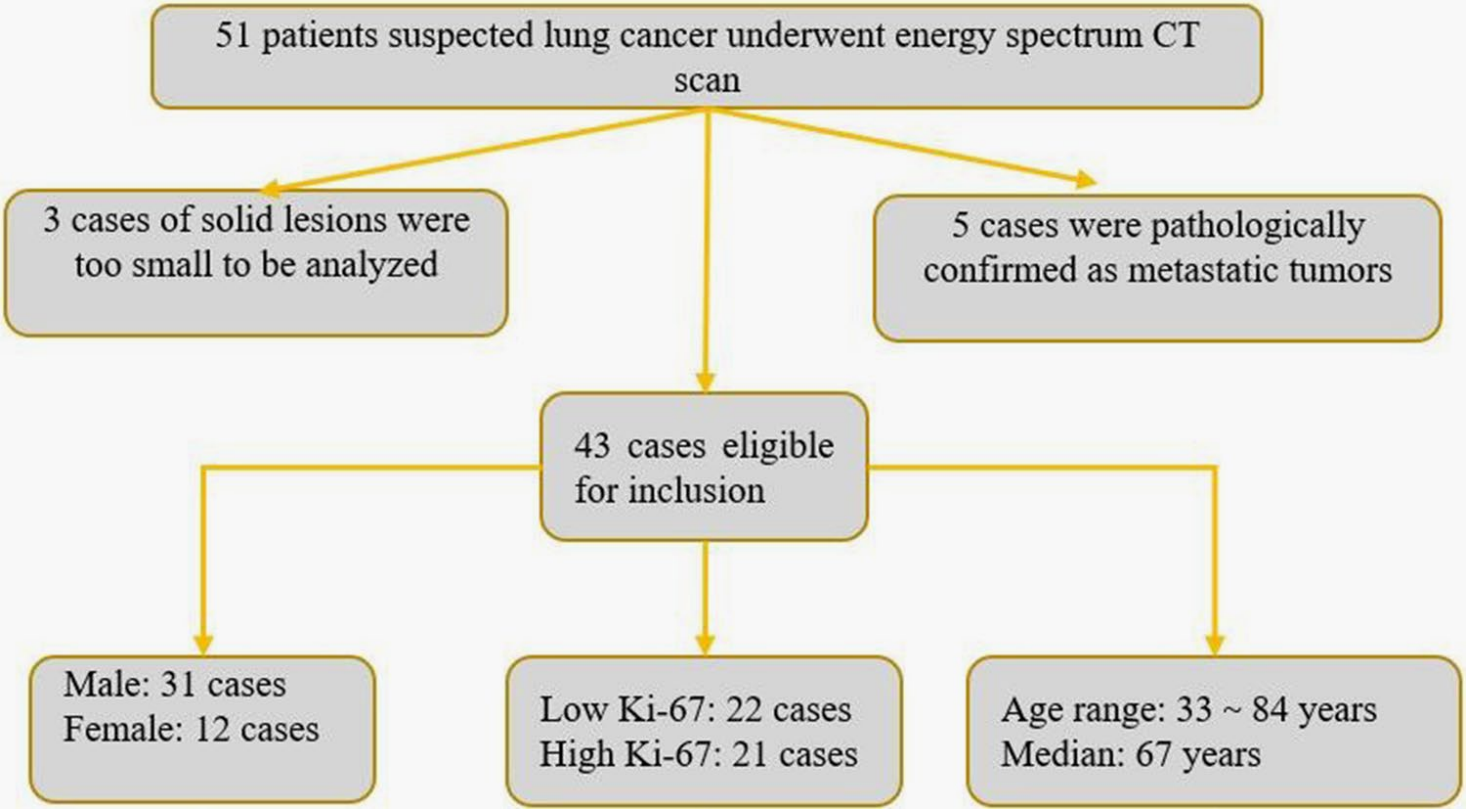
Flow chart of patient selection in this study.

Although fifty-one consecutive patients suspected of having lung cancer were identified between January 2019 and September 2022, only 43 patients were included in this study (Fig. 1).

Inclusion criteria were as follows: Patients (1) cconfirmed with lung cancer by surgical pathology; (2) who did not receive any relevant treatment before undergoing CT examination; (3) who did not undergo Ki-67 immunochemical analysis before; (4) with an interval of dual-energy CT scanning and Ki-67 analysis of less than 2 weeks; and (5) with at least one measurable lesion used for imaging segmentation.

Exclusion criteria were as follows: Patients (1) complicated with other malignancies and has history of any treatment; (2) with benign neoplasm or lung metastasis as confirmed by pathological examination; (3) with incomplete clinicopathological data or missed dual-energy CT examination imaging; and (4) with the quality of images does not meet the needs of this study.

Of the 51 patients, 5 were excluded because of metastatic lung cancer and 3 were excluded because of the solid lesions being too small to allow determination of the region of interest (ROI). All the included 43 patients were first diagnosed and had not received any anti-tumor treatment before dual-energy CT scanning. All the surgeries were performed within 2 weeks of the CT examination.

### Dual-energy CT examination protocol

A dual-phase, dual-energy enhanced chest scan using a Siemens dual-source CT scanner (SOMATOM Force, Siemens Healthcare, Forchheim, Germany) was performed on all patients before treatment. The scanned area spanned from the tip to the base of the lung in the deep inspiration terminal phase. Nonionic iodine contrast agent iodohydrin, comprising 350 mg/mL iodine (GE Healthcare Shanghai Co. Ltd., Shanghai, China), was injected into the median elbow vein at a rate of 2.5 mL/s using a mechanical high-pressure syringe (Ulrich Medical, Missouri, USA) following the standard of 1.0 mL/kg body weight. Then, 20 mL of normal saline was washed at a speed of 3.0 mL/s to reduce the residual contrast agent in the vena cava and reduce the artifacts. Enhanced images of the arterial and venous phases (VP) were obtained after 30 and 75 s of scanning, respectively, after the initial injection. Automatic scanning, grouting, and tracking technology were used. The ROI was set at the proximal descending aorta. The automatic scanning was triggered after a 6-s delay when the trigger threshold reached 100 HU. The dual-energy CT scan parameters included the following: A and B spherical x-ray tube voltages were 90 and 150 kV, respectively, and the reference currents were 77 and 100 mA, respectively. Subsequently, CARE Dose 4D (Siemens) (an automatic exposure-control technology to maximize the reduction of radiation dose without affecting image quality) was switched on. The dual-energy CT scan parameters considered in this study are presented in Table 1.

**Table 1:** Dual-energy CT scan parameters considered in this study.

Parameter	Value
X-ray tube rotation time (s)	0.25
Detector alignment (mm)	192 × 0.6
Pitch (mm)	1.0
Thickness of scanning (mm)	0.5
Space of scanning (mm)	0.5
Thickness of reconstruction (mm)	1.0
Space of reconstruction (mm)	0.5
Convolution kernels	Qr40
Pulmonary window position (HU)	–400
Pulmonary window width (HU)	1500
Mediastinal window position (HU)	40
Mediastinal window width (HU)	400

### Image analysis and data acquisition

**Figure 2:**
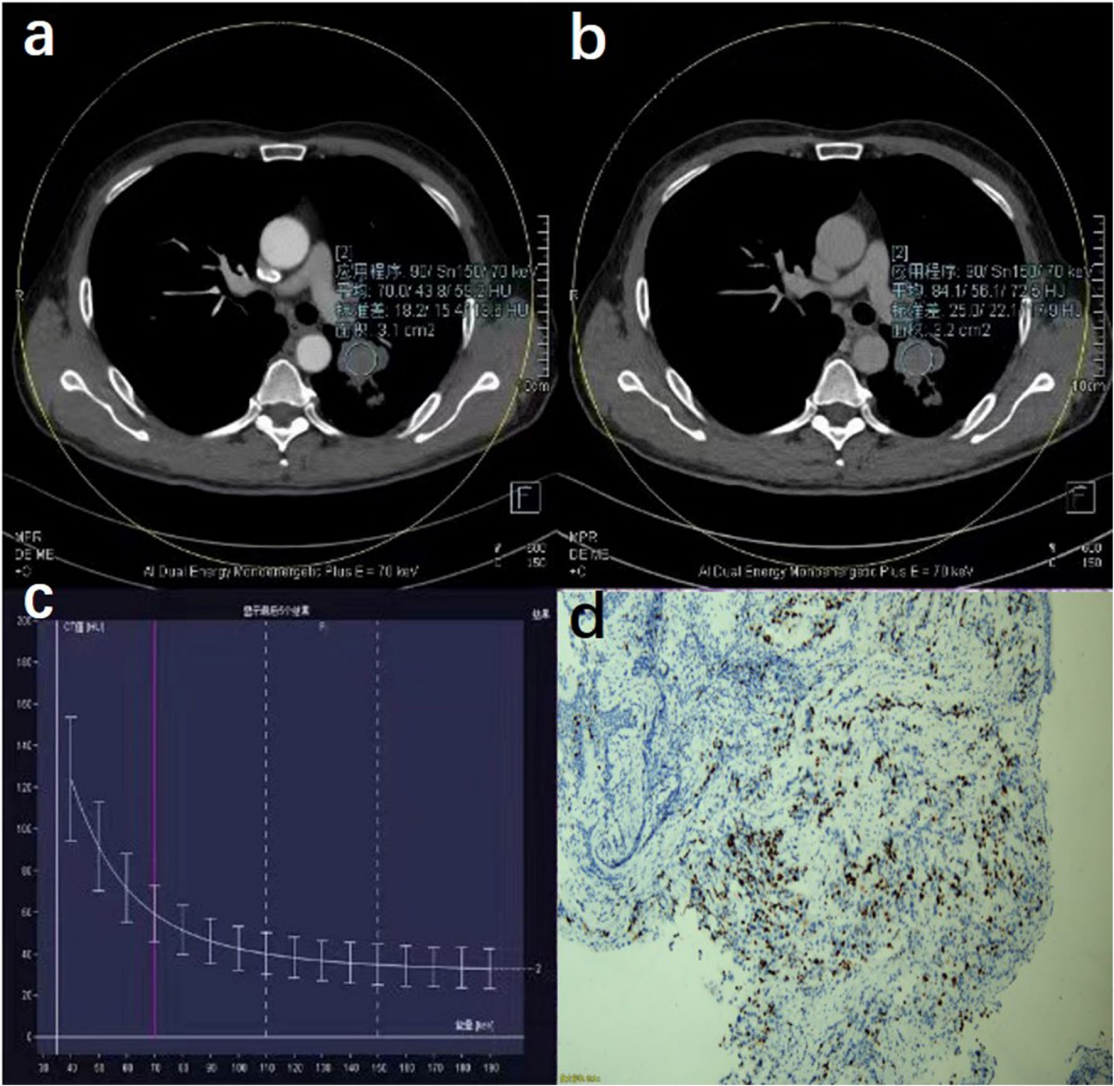
A 60-year-old male patient with lung adenocarcinoma of the left upper lobe (Ki-67 expression, 10%). (**a**) ROI in the arterial phase; (**b**) ROI in the venous phase; (**c**) energy spectrum curve under different single-energy x-ray sequences. (**d**) Hematoxylin and eosin staining with low expression of Ki-67 (10%).

The virtual monochromatic spectral image processing and analysis were performed by two experienced radiologists, respectively. The ROI was manually delineated in the largest tumor layer with reference to the 120-kV mixed-energy image to generate a spectral curve for each lesion. ROI shape was round or oval and comprised the solid part of the lesion as much as possible. Furthermore, necrosis, calcification, visible blood vessel shadow, and gas–liquid plane where artifacts were produced were ignored as much as possible during measurement. The energy spectrum curve of the target lesion was acquired at the processing station (syngo.via VB10, Dual Energy, Siemens, Germany) after drawing the ROI and CT values of 151 single-energy x-ray sequences between 40 and 190 keV of the lesion as automatic output (Fig. 2). The identification of the ROI was done by one radiologist first and then reviewed by another radiologist.

The parameters (longest and shortest diameters) measured by two radiologists were averaged for analysis. The tumor size, necrosis, and ground-glass opacity (GGO) were decided by the two radiologists together. Distant metastases were evaluated by magnetic resonance imaging (MRI) of the brain, abdominal ultrasound, and/or positron emission tomography and CT/MRI examinations. The Ki-67 expression value in each lesion was obtained from the pathological report of our hospital. The median Ki-67 expression index was used for grouping, and the enrolled patients were divided into the Ki-67 high-expression group and the Ki-67 low-expression group.

### Statistical analysis

The CT values of the 11 single-energy x-ray sequences (10-keV intervals) between 40 and 140 keV of the lesion were measured based on the results obtained from each spectral curve. The λHU value of each curve was calculated between 40 and 100 keV [λHU_40–100_ = (CT_40keV_ – CT_100keV_)/(100–40)], 100 and 140 keV [λHU_100–140_ = (CT_100keV_ – CT_140keV_)/(140–100)], and 40 and 140 keV [λHU_100–140_ = (CT_100keV_ – CT_140keV_)/(140–100)]. The spectral data were recorded in Excel for analysis. SPSS Statistics 22.0 was used for statistical analysis. The measurement data were compared using means between the two groups. The normality test and homogeneity of variance test were performed first. If the data were normally distributed and had homogeneous variances, the independent-sample *t* test was used; the Mann–Whitney Utest was used when the variance was not uniform. The qualitative data included the sex, age, tumor size, necrosis, and GGO sign of patients, and χ^2^ test was used to compare the differences in variables. The *P* value < 0.05 indicated a statistically significant difference.

## Results

### Demographic and imaging characteristics

Among the 43 patients with lung cancer, 31 (72.9.4%) were men and 12 (27.1%) were women. Twenty-four (55.8%) patients were aged more than 67 years, and 19 (44.2%) patients were of the age less than or equal to 67 years. A total of 9 (20.9%) and 19 (44.2%) lesions were accompanied by necrosis and GGO, respectively. Nodules and masses were detected in 7 (16.3%) and 36 (83.7%) patients, respectively. A total of 9 (20.9%) resection lesions were small-cell lung cancer, and 34 (79.1%) were non-small-cell lung cancer. The immunochemical analysis revealed that 22 patients (51.2%) had high Ki-67 expression and 21 patients (48.8%) had low Ki-67 expression (Table 2). Ki-67 is a protein that is encoded by the MKI-67 gene in humans.

**Table 2:** Demographic characteristics of enrolled patients (*N* = 43).

Characteristics		*N*	Frequency (%)
Sex	Male	31	72.1
	Female	12	27.9
Age (median, year)	≤ 67	24	55.8
	> 67	19	44.2
Size (cm)	Nodule (≤ 3)	7	16.3
	Mass (>3)	36	83.7
Longest diameter (median, mm)	≤ 50	23	53.5
	> 50	20	46.5
Shortest diameter (median, mm)	≤ 34	22	51.2
	> 34	21	48.8
Ground-glass opacity (GGO)	Yes	19	44.2
	No	24	55.8
Necrosis	Yes	9	20.9
	No	34	79.1
Lymphatic metastasis	Yes	22	51.2
	No	21	48.8
Distant metastasis	Yes	8	18.6
	No	35	81.4
Small-cell lung cancer	Yes	9	20.9
	No	34	79.1
Ki-67 expression (median, %)	Low (≤ 30)	22	51.2
	High (˃30)	21	48.8

### Differences in demographic and imaging features between the high- and low-Ki-67 expression groups

Among the characteristics in 43 patients, only the tumor size was significantly different between the high- and low-Ki-67 expression groups (*P* = 0.048 in the longest diameter and *P* = 0.008 in the shortest diameter). The statistical analysis showed that the high-Ki-67 expression group had larger long or short diameters. Other qualitative parameters, such as sex, age, necrosis, and GGO, did not differ significantly among all enrolled patients, as found using the χ^2^ test (Table 3).

**Table 3:** Differentiation between low- and high-Ki-67 expression groups (*N* = 43).

Characteristic		Ki-67(≤ 30)	Ki-67(>30)	*P*
Sex	Male	14	17	0.355
	Female	8	4	
Age (median, year)	≤ 67	11	13	0.432
	> 67	11	8	
Size (cm)	Nodule (≤ 3)	6	1	0.113
	Mass (> 3)	16	20	
Longest diameter (median, mm)	≤ 50	15	8	**0.048**
	> 50	7	13	
Shortest diameter (median, mm)	≤ 34	14	4	**0.008**
	> 34	8	17	
Ground-glass opacity (GGO)	Yes	7	12	0.095
	No	15	9	
Necrosis	Yes	2	8	0.059
	No	20	13	
Lymphatic metastasis	Yes	10	12	0.558
	No	12	9	
Distant metastasis	Yes	7	1	0.059
	No	15	20	
Small-cell cancer	Yes	2	7	0.114
	No	20	14	

Ki-67 is a protein encoded by the MKI-67 gene in humans. Values in bold indicate low- and high-Ki-67 expression groups having significant differences.

### CT values at different energy levels between high- and low-Ki-67 expression groups

**Figure 3:**
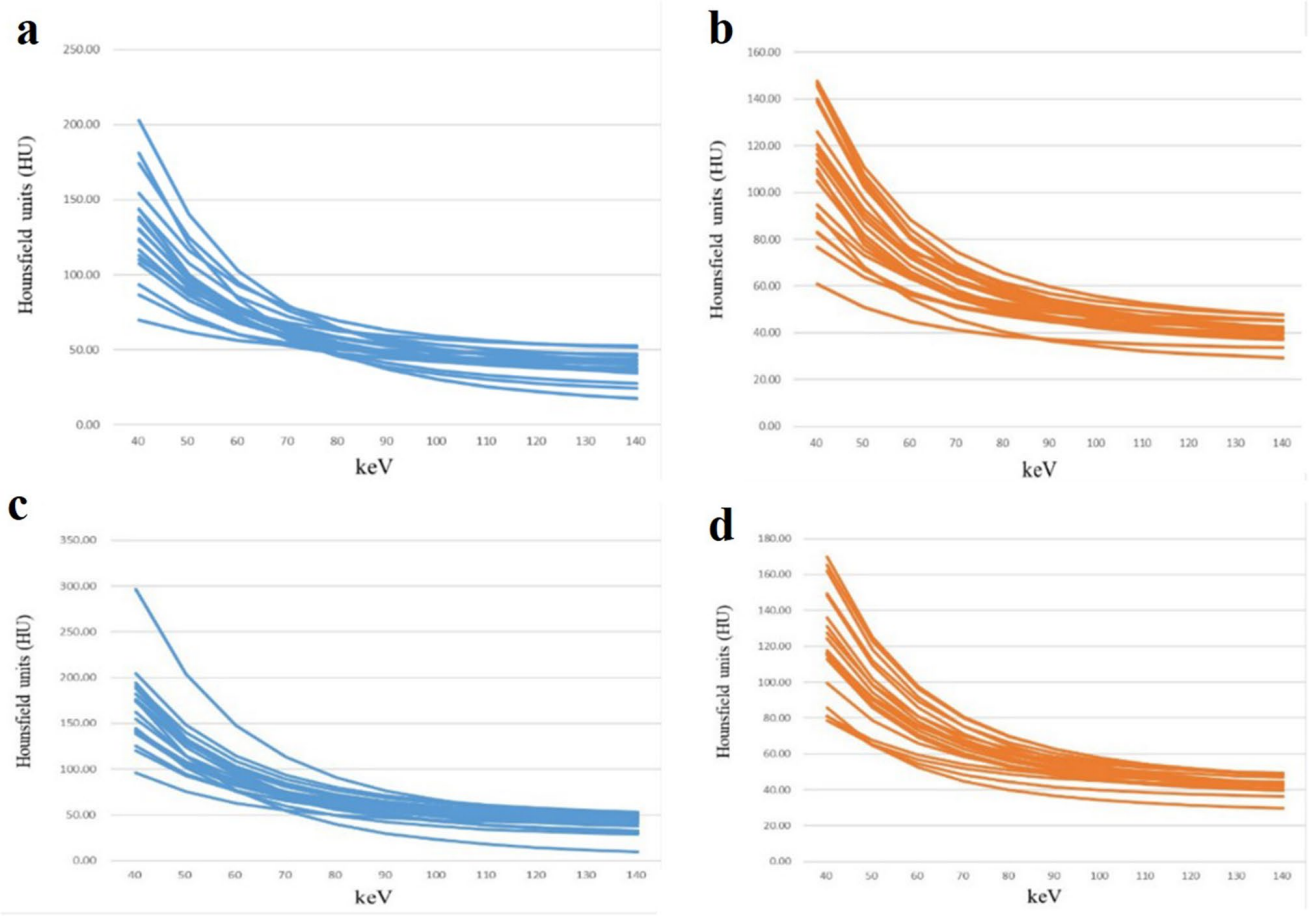
Energy spectrum attenuation curves in the high- and low-Ki-67 expression groups. (**a**) Low-Ki-67 expression group in the arterial phase; (**b**) high-Ki-67 expression group in the arterial phase; (**c**) low-Ki-67 expression group in the venous phase; and (**d**) high-Ki-67 expression group in the venous phase.

**Figure 4:**
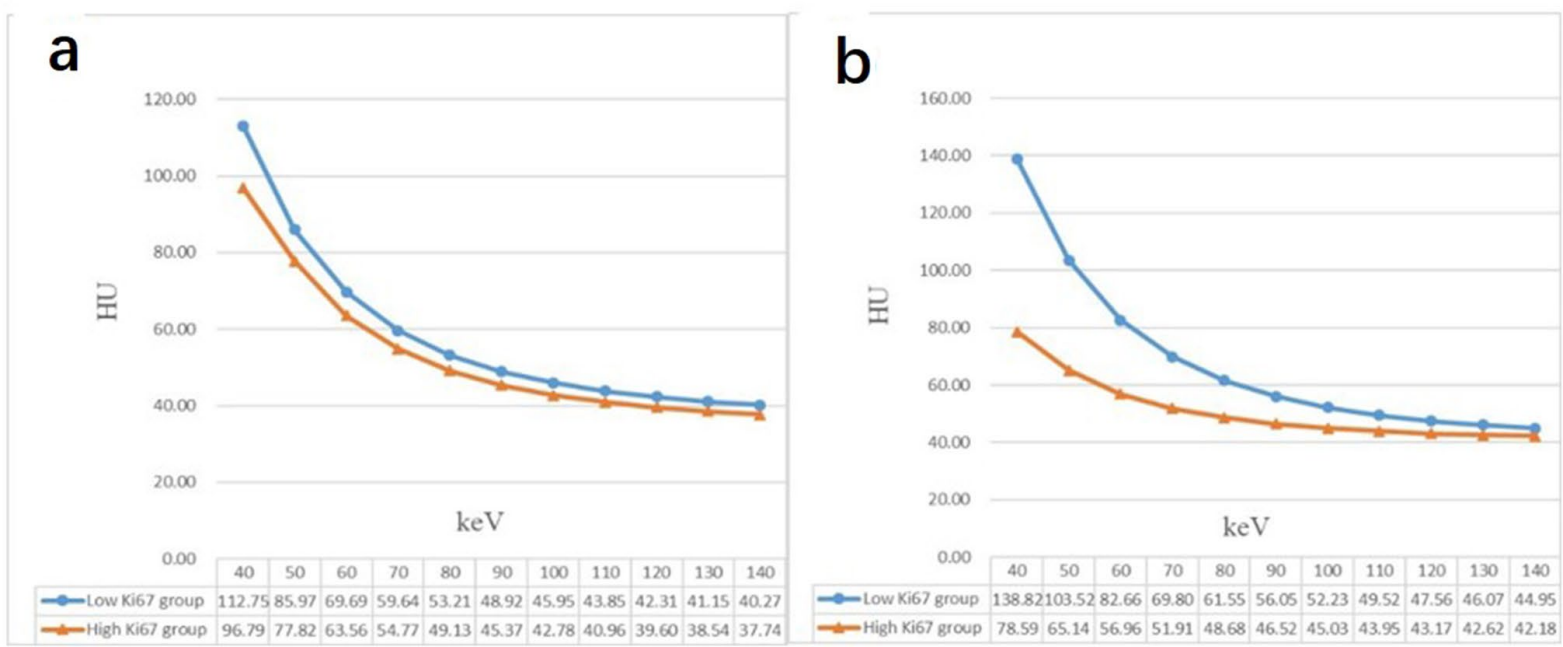
Contrast in energy spectrum attenuation curves in the high- and low-Ki-67 expression groups in the arterial and venous phases: (**a**) arterial phase and (**b**) venous phase.

We measured the CT values of 11 single-energy x-ray sequences (10-keV intervals) between 40 and 140 keV for arterial and VPs by analyzing the attenuation curve of the energy spectrum (Fig. 3). The CT values in the high-Ki-67 expression group was generally lower than that in the low-Ki-67 expression group in both arterial and VPs; the trend was more obvious in the VP and gradually decreased with increase in the energy levels (Fig. 4). Further analysis showed that CT values between 40 and 50 keV were significantly different between the high- and low-Ki-67 expression groups during the arterial phase (AP). CT values corresponding to 40, 60, and 70 keV in the VP were significantly different between the high- and low-Ki-67 expression groups (Supplementary Table 1).

### The difference in λHU value between high- and low-Ki-67 expression groups

**Figure 5:**
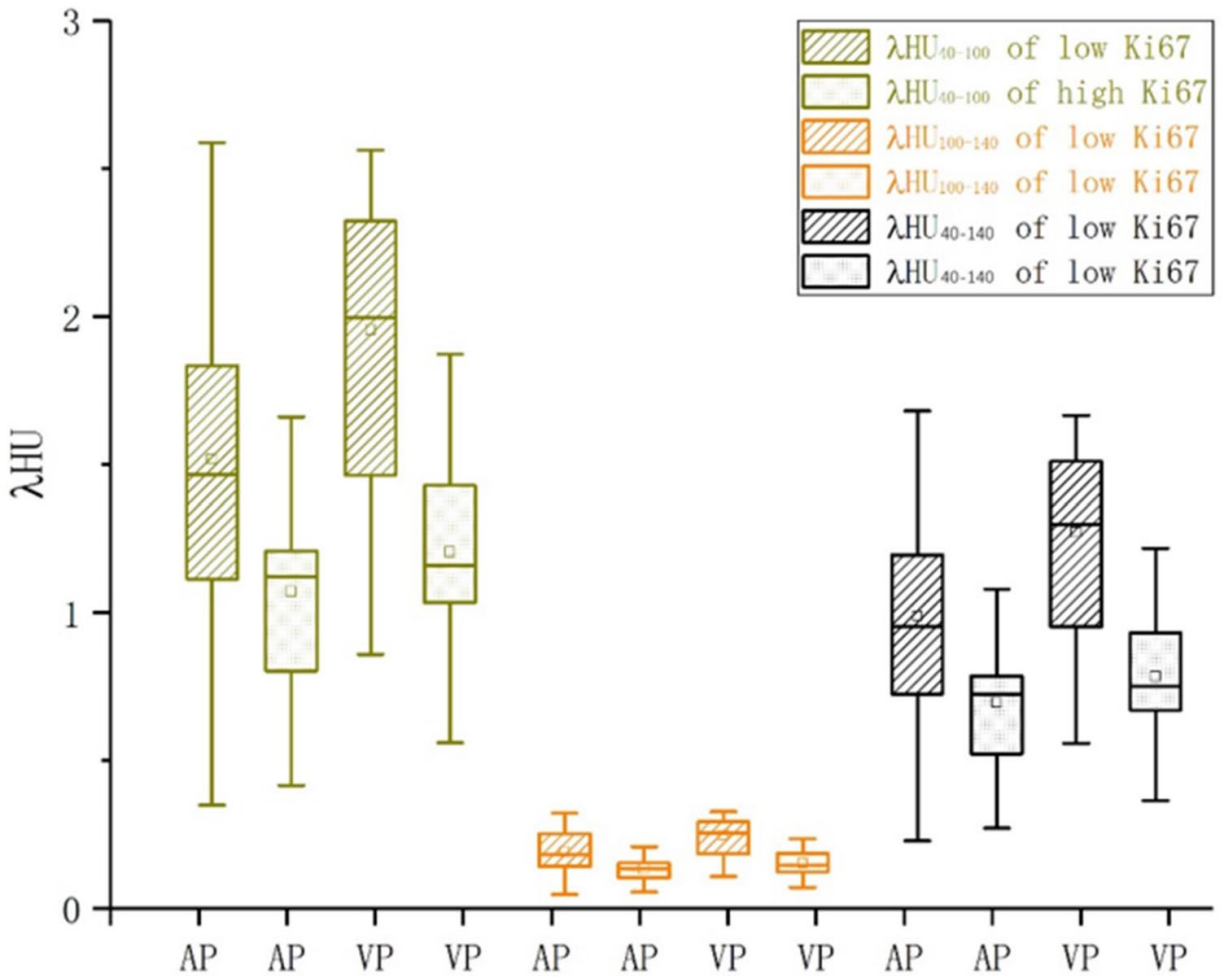
Slope distribution of different segments between high- and low-Ki-67 expression groups.

We observed the attenuation characteristics of the energy spectrum curve and considered three segments of λHU values (λHU_40–100_, λHU_100–140_, and λHU_40–140_) for analysis in the arterial and VPs. After dividing the enrolled patients into two groups based on Ki-67 median value, the λHU values of the three segments in the low-Ki-67 expression group were higher than those in the high-Ki-67 expression group in both arterial and VPs (Fig. 5). Besides, the figure shows that λHU value in the VP was generally higher than that in the AP. Further statistical analysis showed that the λHU value of the three segments was significantly different between the two groups, regardless of the arterial or VP. The significance levels of the slopes of the three segments were identical in the AP (*P* = 0.006) and also in the VP (*P* = 0.000), with all the significance levels less than 0.01 (Table 4).

**Table 4:** Difference in arterial- and venous-phase slopes of the energy spectrum curve in low- and high-Ki-67 expression groups (*N* = 43).

Variables	Low-Ki-67 expression group (± S)	High-Ki-67 expression group (± S)	*P*
AP			
**λ**HU_40–100_	1.518 ± 0.611	1.072 ± 0.364	**0.006**
**λ**HU_100–140_	0.192 ± 0.077	0.134 ± 0.044	**0.006**
**λ**HU_40–140_	0.988 ± 0.397	0.696 ± 0.236	**0.006**
VP			
**λ**HU_40–100_	1.953 ± 0.636	1.206 ± 0.383	**0.000**
**λ**HU_100–140_	0.247 ± 0.081	0.152 ± 0.048	**0.000**
**λ**HU_40–140_	1.271 ± 0.414	0.784 ± 0.249	**0.000**

Values in bold indicate significant differences in low- and high-Ki-67 expression index (*P* < 0.05).

AP = arterial phase; VP = venous phase; λHU_40–100_ = the slope of energy spectrum attenuation curve between 40 and 100 keV; λHU_100–140_ = the slope of energy spectrum attenuation curve between 100 and 140 keV; λHU_40–140_ = the slope of energy spectrum attenuation curve between 40 and 140 keV.

### Performance of slopes of the energy spectrum curve for predicting Ki-67 expression

**Figure 6:**
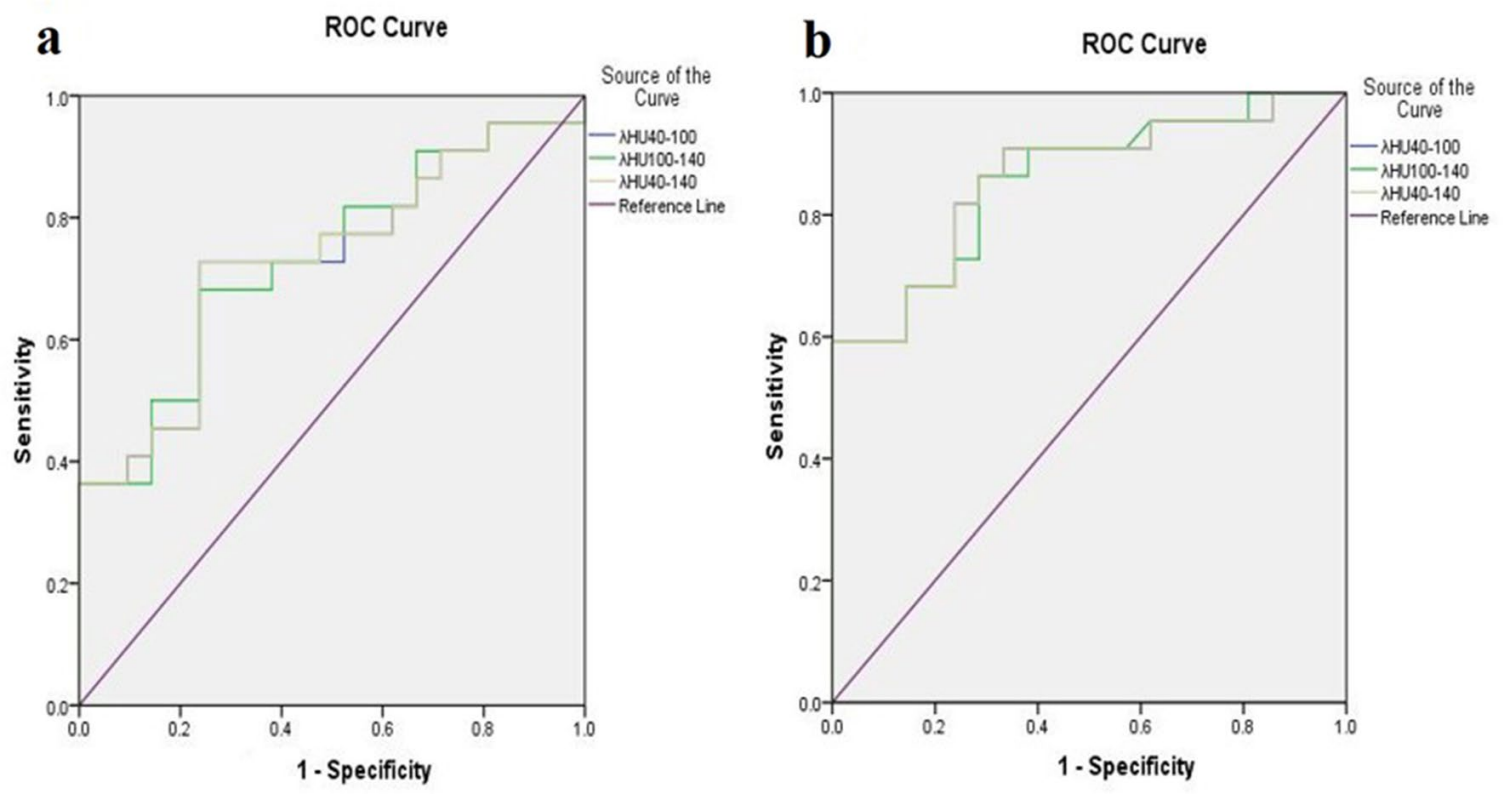
ROC curves of apλHU (**a**) and vpλHU (**b**).

We plotted the receiver operating characteristic (ROC) curve of three λHU segments (λHU_40–100_, λHU_100–140_, and λHU_40–140_) of AP (apλHU) and VPs (apλHU) (Fig. 6). The ROC analysis of apλHU_40–100_ showed borderline *P* value (*P* = 0.004) with areas under the curve (AUC) value of 0.727. The sensitivity (SE), specificity (SP), and cutoff values were 0.717, 92.86, and ≤ 1.212 for apλHU_40–100_, respectively. The ROC analysis of apλHU_100–140_ showed a borderline *P* value (*P* = 0.003) with an AUC value of 0.732. The SE, SP, and cutoff values were 76.19, 68.18, and ≤ 0.154 for apλHU_100-140_, respectively. The ROC analysis of apλHU_40–140_ showed a borderline *P* value (*P* = 0.004) with an AUC value of 0.729. The SE, SP, and cutoff values were 76.19, 72.73, and ≤ 0.788 for apλHU_40–140_, respectively. The ROC analysis of the three λHU segments of the VP showed the same borderline *P* value (*P* = 0.000), SE value of 100, and SP value of 59.09, while their AUC values (0.859 for apλHU_40–100_ and apλHU_40–140_, 0.856 for apλHU_100–140_) and cutoff value (1.892 for apλHU_40–100_, 0.238 for apλHU_100–140_, and 1.230 for apλHU_40–140_) were slightly different (Table 5).

**Table 5:** ROC curve analysis of the energy spectrum CT parameters.

Variables	AUC	Cutoff value	SE(%)	SP(%)	*P*
AP					
λHU40–100	0.727	1.212	76.19	72.73	0.0041
λHU_100–140_	0.732	0.154	76.19	68.18	0.0030
λHU_40–140_	0.729	0.788	76.19	72.73	0.0037
VP					
λHU40–100	0.859	1.892	100	59.09	˂0.0001
λHU100–140	0.856	0.238	100	59.09	˂0.0001
λHU40–140	0.859	1.230	100	59.09	˂0.0001

AP = arterial phase; VP = venous phase; λHU_40–100_ = the slope of energy spectrum attenuation curve between 40 and 100 keV; λHU_100–140_ = the slope of energy spectrum attenuation curve between 100 and 140 keV; λHU_40–140_ = the slope of energy spectrum attenuation curve between 40 and 140 keV.

## Conclusions and discussion

In this study, the slope of the selected three-segment energy spectrum curve was significantly different between the high- and low-Ki-67 expression groups. The predictive efficacy of curve slopes in the VP was better than that in the AP, while the predictive efficacy of the three-segment slopes in the VP was almost the same. The CT values, which were obtained between 40 and 140 keV, in the low-Ki-67 expression group were generally higher than those in the high-Ki-67 expression group in both arterial and VPs. The CT values corresponding to low energy levels showed a huge difference between the high- and low-Ki-67 expression groups, especially in the VP. The difference in CT values between the two groups gradually decreased with the increase in the energy level.

The lesions in the high-Ki-67 expression group had a larger volume, which might be related to Ki-67’s own characteristics. A large number of studies showed that, in malignant tumors, the higher the expression index of Ki-67, the faster the cell proliferation^5,19–21^; therefore, the corresponding malignant mass volume expanded rapidly. Perhaps this factor could explain the larger size of tumors with high Ki-67 expression.

DECT energy spectrum imaging is the postprocessing algorithmic reconstruction of the original energy data obtained under the voltage of two tubes. Its theoretical basis is the principle of material separation, and based on this principle, 151 single-energy images of 40–190 keV can be obtained with corresponding CT values for each single energy level^22^. The characteristics of the CT energy spectrum curve formed by different component tissues are different; therefore, the change in the attenuation of the CT value can be used to reflect different lesions^23–25^. The difference in spectral curves needs to be correlated with the iodine concentration in the lesions when the contrast agent is applied. We can assume that the change in attenuation of the energy spectrum curve is closely related to the blood supply of lesions since the concentration of iodine in lesions indirectly reflects the blood supply of lesions to some extent^26^. In this study, not only the CT values but also the λHU values in all three segments of the curve were higher in the low-Ki-67 expression group than in the high-Ki-67 expression group. The negative association between CT values (as well as λHU) and the degree of Ki-67 expression could be attributed to the presence of intratumoral hypovascularity. The tumor had a relatively insufficient blood supply and increased necrosis^27^ due to the rapid proliferation of tumor cells with high Ki-67 expression^5,19–21^. As a result, the CT value and attenuation amplitude of these lesions were relatively small.

Huijuan et al. found in the study of isolated pulmonary nodules that the λHU values in the energy spectrum curve could well distinguish benign and malignant lung diseases, and the λHU value in the benign group was significantly higher than that in the malignant group^28^. In this study, the λHU values of the selected three-segment energy spectrum curve of the arterial and VPs were significantly different between the high- and low-Ki-67 expression groups. Moreover, the λHU values in the high-Ki-67 expression group were significantly lower than those in the low-Ki-67 expression group. The expression level of Ki-67 indicates the degree of tumor malignancy to a certain extent. Notably, the results of our study and Huijuan’s study were consistent. Besides, we found that the vpλHU values were a better predictor of Ki-67 expression than the apλHU values using the ROC curve analysis. Yang et al. found that the VP energy spectrum parameters had a better predictive value in the study of lymph-node metastasis of non-small-cell lung cancer^29^. This might be because the iodine contrast agent was maximally filled inside the lesion, thus enabling the best contrast and development of the tissue composition inside the lesion in the VP.

In dual-energy imaging, differences in attenuation curves are magnified at different energy levels, allowing differentiation based on different CT values at varying energy levels^30^. In this study, the CT values decreased with the increase in the energy levels in both high- and low-Ki-67 expression groups; the difference between the two groups with low energy levels was significant, and with the increase in the energy level, the difference between the two groups gradually decreased. This result suggested that low energy level was better at distinguishing the differences in internal tissue composition of the lesion, similar to the findings of Jennifer et al. in their study on noncalcified gallstones using dual-energy CT^31^.

This study had some limitations. (1) The sample size was small and hence to improve diagnostic accuracy further studies should consider large sample size. (2) The distribution of lung cancer types was uneven, with adenocarcinoma accounting for the majority, while small-cell lung cancer and squamous cell carcinoma were rare. (3) This study only analyzed the relationship between primary lung cancer and energy spectrum attenuation curve, and did not analyze other pulmonary space–occupying lesions, such as metastatic cancer and benign lesions. (4) The correlation between patient outcomes and findings could not be examined because of the lack of follow-up data.

Further studies should focus more on the diagnostic pathways and clinical benefits of using dual energy quantitatively and perform longer-term follow-ups for each patient.

In conclusion, a correlation between the energy spectrum attenuation curve and the degree of Ki-67 expression was observed in this study, providing valuable information to distinguish lung cancers with low-Ki-67 expression from high-expression Ki-67 lung cancers with high Ki-67 expression. Besides, the use of vpλHU and the analysis of the image at a low energy level may improve the accuracy in differentiating between tumors with low and high Ki-67 expression before subsequent treatments. Understanding the expression level of Ki-67 in lung cancer can guide the treatment to some extent. It may assist in performing spectral CT imaging in patients with lung cancer and obtaining more imaging information about the tumor before a biopsy.

### Ethical approval

The trial was conducted following the Declaration of Helsinki. This study was approved by the ethics committee of the Affiliated Hospital of Xuzhou Medical University (ID: XYFY2018-KL097-01). The informed consent was waived off by the ethics committee of the Affiliated Hospital of Xuzhou Medical University for this study.

## Supplementary Information

Supplementary Information 1.

Supplementary Information 2.

## Data Availability

All data generated or analyzed during this study are included in this manuscript and in the supplementary information files.
