# Supplementary material for: Virtual monoenergetic imaging predicting Ki-67 expression in lung cancer
Source: Sci Rep. 2023 Mar 7;13:3774. doi: 10.1038/s41598-023-30974-5 (PMC9992396; doi:10.1038/s41598-023-30974-5)
Supplement: Supplementary file 2 — Supplementary Information 2. [file 41598_2023_30974_MOESM2_ESM.docx]

**Supplementary Table 1.** Energy levels in the arterial and venous phases in low- and high-Ki-67 expression groups (*N* = 43)

| Variables | Low-Ki-67 (±S) | | High-Ki-67 (±S) | *P* |
| --- | --- | --- | --- | --- |
| AP | |  |  |  |
| 40 keV | | 131.336 ± 31.009 | 110.272 ± 24.912 | **0.019** |
| 50 keV | | 95.251 ± 18.519 | 84.257 ± 16.406 | **0.046** |
| 60 keV | | 72.867 ± 13.807 | 68.438 ± 11.159 | 0.255 |
| 70 keV | | 59.063 ± 13.346 | 59.046 ± 8.417 | 0.996 |
| 80 keV | | 50.214 ± 14.241 | 52.894 ±6.673 | 0.438 |
| 90 keV | | 44.320 ± 15.282 | 48.813 ± 5.682 | 0.208 |
| 100 keV | | 40.247 ± 16.162 | 45.980 ± 5.100 | 0.126 |
| 110 keV | | 37.615 ± 16.994 | 43.975 ± 4.769 | 0.104 |
| 120 keV | | 35.272 ± 17.419 | 42.520 ± 4.575 | 0.072 |
| 130 keV | | 33.738 ± 17.825 | 41.450 ± 4.470 | 0.061 |
| 140 keV | | 32.561 ± 18.135 | 40.636 ± 4.416 | 0.054 |
| VP | |  |  |  |
| 40 keV | | 166.079 ± 39.887 | 121.423 ± 27.285 | **0.000** |
| 50 keV | | 109.563 ± 36.894 | 92.459 ± 18.257 | 0.063 |
| 60 keV | | 90.522 ± 19.710 | 74.799 ± 12.863 | **0.004** |
| 70 keV | | 72.895 ± 16.785 | 63.909 ± 9.658 | **0.039** |
| 80 keV | | 61.595 ± 15.750 | 56.929 ± 7.721 | 0.228 |
| 90 keV | | 54.065 ± 15.514 | 52.303 ± 6.554 | 0.633 |
| 100 keV | | 48.869 ± 15.587 | 49.079 ± 5.800 | 0.953 |
| 110 keV | | 45.173 ± 15.752 | 46.802 ± 5.334 | 0.651 |
| 120 keV | | 42.480 ± 15.932 | 45.153 ± 5.028 | 0.461 |
| 130 keV | | 40.495 ± 16.081 | 43.941 ± 4.820 | 0.346 |
| 140 keV | | 38.987 ± 16.218 | 43.016 ± 4.670 | 0.274 |

Note: Values in bold indicate significant differences in low- and high-Ki-67 expression index (*P* < 0.05).AP = arterial phase, VP = venous phase.
